# Preliminary effects and feasibility of a school-based regular aerobic exercise intervention on PTSD-related symptoms among college students: a single-group pre-post study

**DOI:** 10.3389/fpubh.2026.1742648

**Published:** 2026-02-04

**Authors:** Yuanyuan Jing, Dajun Yang, Yun Ren, Xiong Ke, Yuanyuan Deng, Juan Zhang, Honggang Hui, Yu Yao, Xinling Xie, Ruining Liu, Muhammad Najib Mohamad Alwi

**Affiliations:** 1 School of Management, North Sichuan Medical College, Nanchong, Sichuan, China; 2 Key Laboratory of Digital-Intelligent Disease Surveillance and Health Governance, North Sichuan Medical College, Nanchong, China; 3 School of Graduate Studies, Post Graduate Centre, Management and Science University, Shah Alam, Selangor, Malaysia; 4 School of Laboratory Medicine, North Sichuan Medical College, Nanchong, Sichuan, China; 5 School of Clinical Medicine, North Sichuan Medical College, Nanchong, Sichuan, China; 6 Department of Psychiatry, International Medical School, Management and Science University, Shah Alam, Malaysia

**Keywords:** anxiety, depression, post-traumatic stress disorder, psychological resilience, school-based regular aerobic exercise intervention

## Abstract

**Background:**

College students are considered a high-risk group for post-traumatic stress disorder (PTSD). Existing non-pharmacological interventions, although effective and accessible, still have notable limitations. Regular aerobic exercise, as a potential mental health promotion strategy, requires empirical validation in real campus environments to determine its specific effects on reducing anxiety, depression, and PTSD symptoms, as well as its role in enhancing psychological resilience.

**Method:**

This study employed a single-group pre-test/post-test design and was conducted in August 2025 at a comprehensive university in Sichuan, China. Using convenience sampling, 47 participants with elevated PTSD symptoms were recruited for the “School-Based Regular Aerobic Exercise” intervention trial (operationalized as a 21-Day Exercise Check-In Activity). All participants engaged in 30 min of aerobic exercise daily for 3 weeks. Before and after the intervention, participants completed the PTSD Checklist for DSM-5 (PCL-5), Self-Rating Anxiety Scale (SAS), Self-Rating Depression Scale (SDS), and Connor-Davidson Resilience Scale (CD-RISC).

**Result:**

All 47 participants completed the 21-day intervention. Significant pre- to post-intervention improvements were observed in PTSD symptoms, anxiety, depression, and psychological resilience. Clinical significance analysis showed that 57.45% of participants fell below the PTSD clinical cutoff after the intervention, and 87.23% demonstrated reliable change. Exploratory change-score analyses indicated that improvements in resilience were significantly associated with reductions in anxiety and PTSD symptoms, but not depression.

**Conclusion:**

In a real-world campus setting, this 21-day school-based regular aerobic exercise intervention appears feasible and is associated with improvements in PTSD-related symptoms and psychological resilience. However, because the study lacked randomization and a control group, causal inferences regarding effectiveness and underlying mechanisms cannot be made. Randomized controlled trials with longer follow-up periods and objective activity measures are warranted.

## 1 Introduction

Post-Traumatic Stress Disorder (PTSD) is a severe mental disorder triggered by exposure to traumatic events (1). Its core features include re-experiencing the trauma, avoidance, negative changes in cognition and mood, and increased arousal (2, 3). PTSD exerts long-term and profound negative impacts on an individual’s social functioning, academic achievement, and overall quality of life (4–6). For example, Park et al. (2022) found that community resilience was significantly associated with PTSD symptoms and quality of life among affected individuals.

College students, who are at a critical stage of psychological development (7), face multiple challenges—including academic pressure, interpersonal difficulties, future planning, and potential exposure to traumatic events—making them widely recognized as a high-risk group for PTSD (8). Zhai and Du (9) reported that the prevalence of PTSD diagnoses among American college students increased annually from 2017 to 2022. During the COVID-19 pandemic, the prevalence among Chinese college students reached 25%, with even higher rates observed among students in Africa, Europe, low-to-middle-income countries, and in medical schools (10).

Despite the effective control of COVID-19, ongoing threats such as wars, extreme weather events, and localized infectious disease outbreaks continue to pose serious challenges (11). Importantly, PTSD in college students not only increases the risks of comorbid anxiety and depression but may also lead to severe consequences such as academic disruption (12, 13), substance abuse, and suicidal behavior, thereby imposing substantial burdens on families, educational institutions, and healthcare systems. Thus, developing effective and easily disseminable PTSD intervention strategies is essential for strengthening mental health service systems in higher education.

Although evidence-based interventions for PTSD – such as trauma-focused cognitive behavioral therapy and eye movement desensitization and reprocessing (EMDR) – are highly effective (14, 15), their widespread their widespread implementation on university campuses faces numerous practical barriers. These include a shortage of quality therapists, students’ reluctance to seek professional help due to stigma, high time and financial costs, and uneven geographical distribution of treatment resources (16, 17). For instance, Silvestrini and Chen (18) found that male veteran college students may be less willing to seek help for PTSD due to feelings of shame and distrust.

Pharmacotherapy is also effective but its potential side effects and risk of dependency limit its suitability for student populations (19, 20). Consequently, there is an urgent need for non-pharmacological, non-invasive auxiliary or alternative interventions that are highly accessible, cost-effective, and easily acceptable to college students. The campus environment, as the primary setting for students’ daily life and learning (21), plays an important role in reducing stress and anxiety, enhancing focus and creativity, and promoting physical activity (22, 23). For example, Guo, Wen (24) reported that campus public spaces can significantly improve students’ mental health. However, existing literature lacks systematic evaluations of how such universal campus-based interventions influence core PTSD symptoms and common comorbidities among college students. Moreover, the mechanisms underlying these potential effects remain largely unexplored, representing a significant research gap.

School-based regular aerobic exercise intervention, as a low-cost, highly accessible, and less stigmatized health-promoting behavior, may help address these gaps (25). Such interventions can enhance students’ sense of self-efficacy through mastery experiences, provide positive distraction to interrupt rumination, and create opportunities for social interaction, thereby improving psychological well-being and body image (26). Yet, most prior studies remained theoretical or limited to small-scale preliminary evaluations.

For example, Young-McCaughan, Peterson (27) found that regular aerobic exercise among active-duty military personnel in the U.S. was associated with reductions in PTSD severity over time. Regular aerobic exercise is fundamentally a healthy lifestyle behavior (28), and the present study integrates this approach into the university context. As both a problem-focused and emotion-focused coping strategy, aerobic exercise may reshape individuals’ cognitive appraisals of traumatic events—shifting from catastrophic interpretations to more adaptive processing (29)—thereby reducing negative mood changes and hyperarousal associated with PTSD.

A key advantage of school-based regular aerobic exercise intervention is their high ecological validity, as they directly engage students within their everyday environments, increasing feasibility and willingness to participate (30). Thus, such interventions may alleviate physiological hyperarousal, reduce comorbid anxiety and depression, and enhance psychological resilience in coping with adversity.

Psychological resilience is defined as a dynamic process through which individuals maintain or rapidly restore psychological functioning when facing adversity, trauma, or stress (31, 32). According to conservation of resources theory (33), traumatic events often deplete of individuals’ psychological resources, triggering a vicious cycle that exacerbates PTSD (34).

Regular aerobic exercise, as a resource-acquisition strategy, may enhance psychological resilience by enriching individual’s resource pools. School-based regular aerobic exercise interventions can strengthen core dimensions of resilience—such as emotional regulation and problem-solving abilities (35) – thereby increasing engagement in exercise and fostering hope for PTSD improvement. Additionally, the sense of achievement and social interaction derived from regular exercise may further translate into psychological resilience, amplifying the intervention effects through positive feedback loops and creating a virtuous cycle (36). Exercise promotes adaptive meaning-making in the face of adversity, enhances resilience, and accelerates post-trauma recovery (37). Based on this theoretical foundation, the present study hypothesizes that psychological resilience plays a significant mediating role between regular aerobic exercise and PTSD symptoms.

Anxiety, as a common comorbidity of PTSD, is characterized by persistent worry and physiological arousal (38). Cognitive-behavioral models suggest that regular aerobic exercise can interrupt anxiety-related rumination and facilitate emotional regulation (39). Aerobic exercise may redirect students’ attention away from anxiety triggers, enhance social integration, and boost self-efficacy and confidence, thereby reducing anxiety stemming from self-doubt. Psychological resilience also plays a protective role in alleviating anxiety symptoms (40). Higher resilience can reduce distress associated with traumatic memories (41) by enabling cognitive reappraisal, decreasing sensitivity to trauma cues, and reducing the frequency and intensity of intrusive memories. Depression, the most common comorbidity of anxiety, often manifests as low mood, anhedonia, and cognitive distortions, and is frequently associated with hormonal changes and environmental stressors during adolescence (42). According to cognitive-emotional interaction theory (43), regular exercise can alleviate depressive symptoms by interrupting cycles of depressive rumination. School-based regular aerobic exercise interventions may counteract anhedonia by enhancing dopamine activity, thereby indirectly reducing avoidance and emotional numbness associated with depression.

Therefore, the present study aimed to provide preliminary, ecologically valid evidence on the feasibility and short-term symptom changes associated with a school-based regular aerobic exercise intervention among college students with elevated PTSD symptoms. We further examined whether changes in psychological resilience were statistically associated with changes in PTSD, anxiety, and depression as an exploratory test of a hypothesized mechanism. Given the single-group pre-post design, these analyses are intended to generate mechanistic hypotheses and inform subsequent randomized controlled trials rather than to establish causal efficacy. The significance and potential contributions of this study is multifaceted. Practically, it offers college administrators and mental health practitioners a specific, feasible, and easily implementable non-pharmacological intervention that can be integrated into existing campus health promotion programs, thereby expanding prevention and treatment options for PTSD and improving the accessibility of campus mental health services. Finally, the study provides foundational data and decision-making support for promoting the formal incorporation of regular physical activity into public mental health service systems for college students in China as a scientifically grounded primary prevention and secondary intervention strategy.

## 2 Method

This study employed a single-group pre-post design in a naturalistic campus setting to assess the feasibility of implementing a school-based regular aerobic exercise intervention and to quantify within-participant changes in PTSD symptoms, common comorbid symptoms (anxiety and depression), and psychological resilience. Because no control group was included, the study was intended as a preliminary feasibility and proof-of-concept investigation rather than a causal efficacy trial.

### 2.1 Participants and sample

This study commenced in May and was completed in its entirety on August 25, at a comprehensive university in Sichuan Province, encompassing the following phases: protocol preparation, participant recruitment and informed consent, intervention implementation, questionnaire/scale assessments, and data collection. The university is well-equipped with sports facilities, exercise venues, and professional sports instructors, meeting the basic requirements for the trial. Participants were recruited using convenience sampling.

PTSD symptom status was operationalized using the PCL-5. Participants were not clinically diagnosed with PTSD for the purposes of this study; instead, eligibility was based on a screening cutoff, which indicates elevated or probable PTSD symptomatology in prior research. Accordingly, we refer to the sample as college students with elevated PTSD symptoms rather than PTSD patients.

The inclusion criteria were: (1) Full-time college students aged 18–25; (2) A preliminary screening for PTSD using the PTSD Checklist 5 (PCL-5) (score ≥ 53, indicating PTSD symptoms); (3) Voluntary participation in the study and signing of an informed consent form; (4) No severe cognitive impairments and sufficient proficiency in Chinese for effective communication; (5) Physical condition permitting normal exercise.

Exclusion criteria were: (1) Severe exercise injuries or illnesses occurring during the study; (2) Failure to complete pre-and post-intervention assessments; (3) Current engagement in other systematic psychological or pharmacological treatments.

### 2.2 Minimum sample size

The minimum sample size was calculated using G*Power software (3.1). This study employed paired sample t-tests with a significance level (*α*) set at 0.05 and an effect size set at 0.5. To achieve a statistical power of 80% (1-*β*), at least 34 participants were needed. Considering a dropout rate of 15%, the study planned to recruit no fewer than 40 participants.

### 2.3 Ethical considerations

The study received approval from the Academic Ethics Committee of North Sichuan Medical College (No.:2025020). All participants were informed of the activity’s purpose, procedures, and reward mechanisms, and signed written informed consent before the study began. They were informed that they could withdraw at any time unconditionally. The research process strictly adhered to confidentiality principles, and data were processed anonymously.

### 2.4 Measurement tools

#### 2.4.1 PTSD checklist

The PCL-5 is a 20-item self-report measure that assesses the presence and severity of PTSD symptoms according to DSM-5 criteria (44). Items are rated on a 5-point Likert scale ranging from 0 (not at all) to 4 (extremely), yielding total scores from 0 to 80. Higher scores indicate greater PTSD symptom severity. Consistent with recommendations from the National Center for PTSD, a cutoff score of 33 is suggested for probable PTSD diagnosis in community samples (45).

In the present study, the scale was translated into Chinese by Su, Kung (46) and validated for cultural adaptation and reliability in five different Chinese populations. A 5-point Likert scale was used for scoring, based on the frequency and severity of symptoms (1 = not at all, 5 = extremely). The total score ranges from 20 to 100 points. Each item score ranges from 1 to 5. In this study, the Cronbach’s alpha for the pre-test and post-test was 0.939 and 0.978, indicating good internal consistency.

#### 2.4.2 Resilience scale

The resilience measurement items were derived from the Connor-Davidson Resilience Scale (CD-RISC) developed by Connor and Davidson (47). This scale consists of 25 items across five dimensions: ability, tolerance of negative emotions, acceptance of change, control, and spiritual influence. The scale was translated into Chinese by Yu and Zhang (48) and validated for cultural adaptation in various populations, such as teachers (49) and medical students (50). A 5-point Likert scale was used for scoring, ranging from 1 = strongly disagree to 5 = strongly agree. Higher scores indicate greater psychological resilience among college students. In this study, the Cronbach’s alpha for the pre-test and post-test was 0.943 and 0.957, indicating good internal consistency.

Higher resilience scores reflect a constellation of adaptive capacities including personal competence and tenacity, tolerance of negative affect and stress, positive acceptance of change, sense of control, and spiritual influences (47). Individuals with higher resilience scores typically demonstrate greater ability to recover from setbacks, maintain psychological equilibrium during stressful circumstances, and adapt positively following adverse experiences.

#### 2.4.3 Depression scale

The depression measurement items were derived from the Zung Self-Rating Depression Scale (SDS) developed by Zung (51). This scale consists of 20 items across three dimensions: pervasive affect, physiological equivalents or concomitants, and psychological concomitants. The scale was translated into Chinese by Cheung (52), and its cultural adaptation and reliability were validated. This study used the scale to measure depression symptoms among college students, employing a 5-point Likert scale for scoring (1 = strongly disagree, 5 = strongly agree). The depression severity index = total score/100, with scores below 0.60 indicating no depression, 0.60–0.72 indicating mild to moderate depression, 0.72–0.84 indicating moderate to severe depression, and above 0.84 indicating severe depression. Higher scores indicate greater depression levels. In this study, the Cronbach’s alpha for the depression scale was 0.919 and 0.815, indicating good internal consistency.

#### 2.4.4 Anxiety scale

The anxiety measurement items were derived from the Zung Self-Rating Anxiety Scale (SAS) developed by Zung (53). This scale consists of 20 items across two dimensions: emotional symptoms and somatic symptoms. The scale was translated into Chinese by Wang and Chi (54) and validated for cultural adaptation and reliability. This study used the scale to measure anxiety symptoms among college students, employing a 5-point Likert scale for scoring (1 = strongly disagree, 5 = strongly agree). The anxiety severity index = total score/100, with scores below 0.60 indicating no anxiety, 0.60–0.72 indicating mild to moderate anxiety, 0.72–0.84 indicating moderate to severe anxiety, and above 0.84 indicating severe anxiety. Higher scores indicate greater anxiety symptom severity. In this study, the Cronbach’s alpha for the anxiety scale was 0.934 and 0.953, indicating good internal consistency.

### 2.5 Intervention process

The pre-test was conducted before the intervention began, and the post-test was completed within 1 week after the 21-day activity ended. The intervention scheme adopted a campus activity format, implementing a “21-Day Exercise Check-In Activity” lasting 3 weeks. Participants were required to choose from three types of exercise: (1) Jump rope for ≥10 min (daily submission of jump rope videos); (2) Run ≥2 kilometers (daily submission of screenshots from the Keep App); (3) Complete a full set of Tai Chi movements (daily submission of a full-length video). All submitted materials (videos or screenshots) must display time information, and forgery is prohibited. Researchers and campus activity staff conducted random on-site checks to ensure authenticity. Participants had to complete 21 consecutive days to qualify for inclusion in the study. Given the relatively large amount of exercise in 30 min, the study required only 30 min of exercise over a single day, and the duration of each exercise session was recorded, amounting to 30 min over a single day.

Participants who completed all assessments (pre-test, post-test) and maintained ≥ 80% exercise session adherence received a gift card valued at approximately ¥50 (approximately $7 USD) for a popular online shopping platform.

#### 2.5.1 Exercise intensity monitoring

Participants recorded their daily exercise sessions using the Keep mobile application and submitted screenshots of the Keep activity logs during daily check-ins. These screenshots were used to document session completion and basic training parameters available in the app. The online platform incorporated daily exercise logs where participants recorded exercise type, duration, and self-rated intensity immediately following each session. Facilitators reviewed these logs and provided individualized feedback within 24 h, including encouragement to adjust intensity if reported levels fell consistently outside target ranges.

#### 2.5.2 Exercise mode flexibility

Participants were permitted flexibility in exercise mode selection within the prescribed framework. At enrollment, participants indicated their preferred primary exercise mode (jump rope, running, or Tai Chi) based on personal preference, available space, equipment access, and physical capacity. However, participants were explicitly informed that they could switch between modes on different days according to circumstances such as weather conditions (e.g., choosing indoor jump rope or Tai Chi instead of outdoor running during inclement weather), physical state (e.g., selecting lower-intensity Tai Chi on days of fatigue or minor muscle soreness), equipment availability, or personal preference on a given day.

The only requirement was that participants complete at least 30 min of one of the three prescribed exercise types daily. Participants were encouraged to maintain consistency where possible to facilitate skill development and routine establishment, but rigid adherence to a single mode was not required. Daily exercise logs captured the specific mode performed each day, allowing for tracking of mode switching patterns.

#### 2.5.3 Structured warm-up, cool-down

All exercise sessions followed a standardized structure consisting of three phases: warm-up, main exercise, and cool-down.

Warm-up Phase (5 min). Each session began with a guided warm-up sequence delivered via pre-recorded video accessible through the online platform. The warm-up included light cardiovascular activation (marching in place, arm circles) and dynamic stretching targeting major muscle groups (leg swings, torso rotations, shoulder rolls, ankle circles). Participants were instructed to complete the warm-up video before every exercise session, regardless of chosen exercise mode.

Main Exercise Phase (30 min). Participants engaged in their selected exercise mode (jump rope, running, or Tai Chi) at the prescribed intensity levels described above.

Cool-down Phase (5 min). Each session concluded with a guided cool-down sequence, also delivered via pre-recorded video, consisting of gradual cardiovascular deceleration (slow walking in place), static stretching for major muscle groups (quadriceps, hamstrings, calves, shoulders, back), and brief breathing exercises to facilitate transition to rest.

#### 2.5.4 Tai Chi instruction

Participants selecting Tai Chi as their primary or supplementary exercise mode received structured instruction through a comprehensive video-based curriculum developed specifically for this intervention.

A 20-min instructional video covering the history and principles of Tai Chi, basic stances (Wuji standing, bow stance, empty stance), fundamental movement principles (weight shifting, coordinated breathing), and safety considerations.

A simplified 8-movement Yang-style Tai Chi form, selected for accessibility and appropriateness for beginners, was taught progressively across the first week. Each movement was demonstrated from multiple angles with detailed verbal instruction regarding posture, weight distribution, hand positioning, and breathing coordination.

Following the instructional phase, participants accessed daily practice videos that guided them through the complete warm-up, Tai Chi form practice (with the form repeated multiple times to fill the 30-min main exercise period), and cool-down. Videos included real-time verbal cues for movement timing, breathing, and common error correction.

#### 2.5.5 Fidelity monitoring

Participants completed structured logs immediately following each session, recording exercise mode, duration, and any difficulties encountered. Log completion was monitored daily, with automated reminders sent to participants who had not submitted logs by 9:00 p.m. Facilitators completed weekly fidelity checklists documenting completion of all scheduled check-ins, response rates to participant logs, and any protocol deviations.

Schematic of the daily exercise structure and the weekly intervention components, as shown in Table 1.

**Table 1:** Schematic overview of daily exercise session structure and weekly intervention components.

Daily session structure (40 min)		
Warm-up (5 Min)	Main exercise(30 Min)	Cool-down (5 Min)
Video-guided Light cardio Dynamic stretching	Option A: Jump rope Option B: Running Option C: Tai Chi	Video-guided Slow walking Static stretching Breathing exercise

Weekly intervention components
Daily: Complete 40-min exercise session (warm-up + exercise + cool-down) Submit exercise log (mode, duration, difficulties) Receive facilitator feedback on log (within 24 h) Weekly: Individual video check-in with facilitator (5–10 min) Review of weekly progress and troubleshooting Form verification for Tai Chi participants (when feasible) As Needed: Additional outreach for participants with concerning patterns Access to all instructional videos for review

21-day intervention timeline		
Week 1 (Days 1–7)	Week 2 (Days 8–14)	Week 3 (Days 15–21)
Orientation Tai Chi intro module Establish routine Week 1 check-in	Continued practice Progressive elements introduced Week 2 check-in	Continued practice Progressive intensity Final check-in Post-test assessment

### 2.6 Data collection and analysis

Data included the results from pre- and post-assessment measures and the 21-day check-in records, all collected and coded by the research team. Statistical analyses were performed using SPSS 26.0. Descriptive statistics were used to analyze demographic characteristics. For differences between pre- and post-assessments, paired sample *t*-tests were applied if the data met normality assumptions; otherwise, Wilcoxon signed-rank tests were used.

Effect size estimation and confidence intervals. For paired-samples t tests, we reported Cohen’s *d_z_* as the standardized mean change, calculated as *d_z_ = t/√N* (equivalently, mean of the paired differences divided by the SD of the paired differences). For Wilcoxon signed-rank tests, we reported the effect size *r = Z/√N*, where N is the number of non-zero paired differences. Ninety-five percent confidence intervals (CIs) for r were estimated using percentile bootstrap resampling (5,000 iterations) of paired observations (no continuity correction). In cases where the bootstrap distribution showed negligible variability, the percentile CI could collapse to a single value.

Change-score (within-person) analytic strategy. Given the single-group pre–post design, observations are not independent if pre- and post-test records are stacked at the measurement level. PROCESS Model 4 is a single-level ordinary least squares (OLS) mediation procedure and does not inherently account for within-subject clustering. Therefore, to avoid violating the independence assumption and to provide a transparent exploratory test of the hypothesized mechanism, we conducted analyses using within-person change scores. For each participant, we computed change scores as *Δ* = post -pre for PTSD, anxiety, depression, and psychological resilience. We then examined whether changes in psychological resilience were associated with changes in PTSD/anxiety/depression using linear regression models (with 95% confidence intervals).

Given the exploratory nature of these analyses and the absence of a control group, results should be interpreted as preliminary evidence of potential mechanisms rather than confirmatory tests of causal mediation. The statistical significance level was set at *p* < 0.05.

## 3 Results

**Figure 1:**
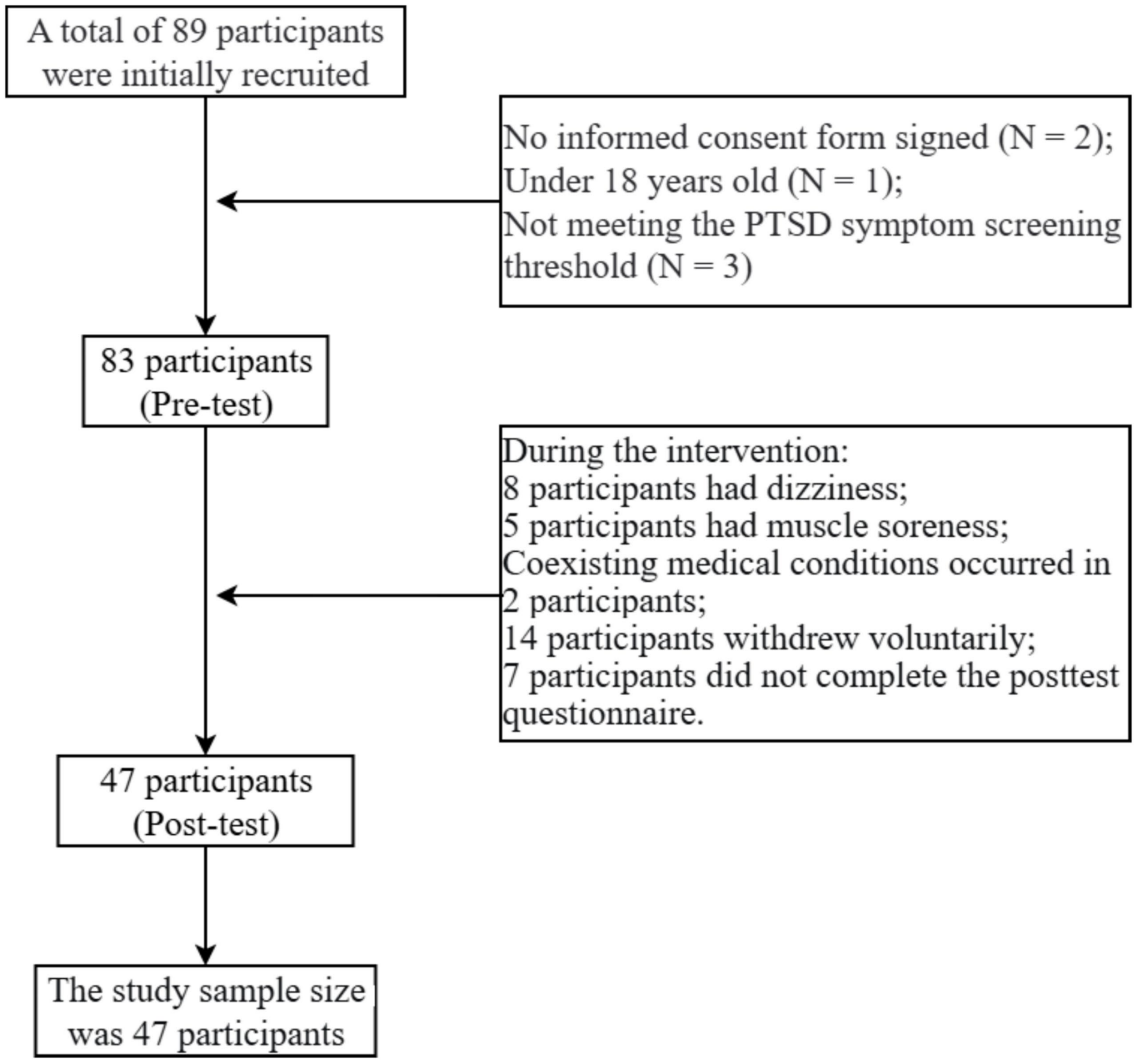
Attrition rate of participants.

A total of 89 students were initially recruited and screened. Thirteen were excluded prior to baseline assessment (2 did not sign informed consent, 1 were younger than 18 years, and 3 did not meet the PTSD symptom screening threshold on the PTSD Checklist), yielding 83 participants who completed the pre-test assessment. During the 21-day program and follow-up assessment period, 36 participants were excluded from the post-test analytic sample: 15 reported physical discomfort (e.g., dizziness or muscle soreness) during the intervention period, 14 withdrew voluntarily, and 7 did not complete the post-test questionnaire. Therefore, 47 participants (24 males, 23 females) completed both pre- and post-assessments and comprised the final analytic sample. The retention rate was 56.62% (47/83) from baseline to post-test and 52.8% (47/89) from initial recruitment to final analysis. Specific demographic information is presented in Table 2. The attrition rate of participants is shown in Figure 1.

**Table 2:** Summary of demographic information.

Demographic dimensions	Specific category	Frequency	Percentage
Gender	Male	24	51.06%
	Female	23	48.94%
Origin of student	Rural	12	25.53%
	Town	35	74.47%
Annual income	Below 100,000 RMB	23	48.94%
	100,000 to 200,000 RMB	17	36.17%
	200,000 RMB and above	7	14.89%
Student grade level	Class of 2022	6	12.77%
	Class of 2023	15	25.53%
	Class of 2024	26	55.32%
During the past 3 weeks of exercise, what are the actual days of exercise for your favorite sport?	10 days or less	31	65.96%
	11–20 days	8	17.02%
	21 days or more	8	17.02%
Exercise duration of single exercise	Less than 30 min	30	63.83%
	30–45 min	10	21.28%
	More than 45 min	7	14.89%
Exercise intensity	Easy	29	61.70%
	Moderate to difficult	18	38.30%
Exercise period	Morning (6–12 a.m.)	4	8.51%
	Afternoon (12–6 p.m.)	3	6.38%
	Evening (6–8 p.m.)	13	27.66%
	Night (after 8 p.m.)	20	42.55%
	Not fixed	7	14.89%
Sports environment	School playground/track	36	76.60%
	Indoor (Dormitory/Gym)	5	10.64%
	Outdoor (Park/Paths)	6	12.77%
Psychological perceptions of exercise	Negative/tried	25	53.19%
	Positive feelings	22	46.81%
Type of exercise	Running	30	63.83%
	Jumping rope	2	4.26%
	Tai Chi	3	6.38%
	Other	12	25.53%

NB: According to the study design, participants were required to complete daily aerobic exercises lasting approximately 30 min per session.

### 3.1 Attrition and potential selection bias

Attrition from baseline (*n* = 76) to the final analytic sample was 38.2% (*n* = 47). Reasons for post-baseline attrition included reported physical discomfort during the intervention (*n* = 15), voluntary withdrawal (*n* = 7), and missing post-test questionnaires (*n* = 7). Because attrition may not be random, the observed pre–post changes should be interpreted cautiously as they may over- or under-estimate symptom changes in the initially recruited population.

Of the 36 non-completers, 15 participants withdrew specifically due to adverse physical symptoms during the intervention sessions. Reported symptoms included dizziness (*n* = 8), muscle soreness (*n* = 5), and combined symptoms (*n* = 2). These symptom-related dropouts occurred predominantly during the initial intervention period (Week 1: *n* = 9; Week 2: *n* = 4; Week 3: *n* = 2), suggesting that early exercise sessions posed the greatest challenge for vulnerable participants.

Informal post-withdrawal contact with these participants revealed that symptoms were typically experienced during or immediately after higher-intensity aerobic segments. Importantly, no participants required medical attention, and all reported symptom resolution within 24–48 h of discontinuation. Nevertheless, the concentration of symptom-related dropout among participants with elevated baseline psychopathology raises concerns about the tolerability of the exercise protocol for more severely affected individuals.

### 3.2 Normality test

We calculated the differences in scores for PTSD, anxiety, depression, and psychological resilience for both pre-test and post-test, followed by exploratory analysis to test for normality. Results indicated that anxiety (*p* = 0.295) and depression (*p* = 0.051) exhibited significant normality, while psychological resilience (*p* = 0.026) and PTSD (*p* < 0.001) showed non-normality. Thus, paired sample t-tests were used for anxiety and depression, whereas Wilcoxon signed-rank tests were applied for psychological resilience and PTSD.

### 3.3 Baseline comparisons between completers and non-completers

We conducted independent samples *t*-tests to compare baseline characteristics between participants who completed the study (*n* = 47) and those who dropped out (*n* = 36).

As shown in Table 3, significant differences emerged between completers and non-completers on all baseline measures (all *p* < 0.001). Notably, participants who dropped out exhibited significantly higher levels of PTSD symptoms, anxiety, and depression, as well as significantly lower psychological resilience, compared to those who completed the intervention. These effect sizes range from medium to large, indicating substantial and clinically meaningful baseline differences between the two groups.

**Table 3:** Baseline comparisons between completers and non-completers.

Variables	Completers (*N* = 47)	Non-completers (*N* = 36)	*t*	*p*	Conhen’s *d*	95% CI
PTSD (M ± SD)	2.404 ± 0.855	4.044 ± 0.700	9.350	<0.001	0.792	[1.528,2.605]
Anxiety (M ± SD)	2.648 ± 0.736	3.568 ± 0.714	5.745	<0.001	0.726	[0.788,1.740]
Depression (M ± SD)	3.087 ± 0.383	3.596 ± 0.637	4.514	<0.001	0.508	[0.537,1.458]
Psychological resilience (M ± SD)	3.584 ± 0.637	2.857 ± 0.723	−5.278	<0.001	0.623	[−1.636,-0.696]

These findings have important implications for interpreting the study results. The pattern suggests that participants with more severe symptomatology and lower resilience resources were disproportionately likely to discontinue participation. This differential attrition may have resulted in a final sample that was less symptomatic and more psychologically resilient than the initially enrolled population, potentially inflating observed treatment effects and limiting generalizability to more severely affected individuals.

### 3.4 Single group pre-and post-test analysis

To further examine the intervention effects of school-based regular aerobic exercise intervention on PTSD, anxiety, depression, and psychological resilience, paired sample t-tests were conducted on anxiety and depression scores pre- (T1) and post-intervention (T2) for all participants, while Wilcoxon signed-rank tests were used for psychological resilience and PTSD. Effect sizes were measured using Cohen’s *d*, with Cohen’s (1988) standards indicating *d* = 0.2 as a small effect, *d* = 0.5 as a medium effect, and *d* = 0.8 as a large effect, as detailed in Table 4.

**Table 4:** Single-group pre- and post-test analysis results.

Variable	T1 Mean	T1 SD	T2 Mean	T2 SD	Δ Mean	Lower limit	Upper limit	T/Z	*p*	Cohen’s *d/r*	95% CI
Anxiety	3.489	0.718	2.648	0.736	−0.842	−1.109	−0.575	−6.352	<0.001	−0.926	[−1.266, −0.580]
Depression	3.514	0.647	3.087	0.383	−0.427	−0.643	−0.209	−3.963	<0.001	−0.578	[−0.885,–0.266]
Psychological resilience	2.843	0.691	3.585	0.534	0.742	–	–	4.842	<0.001	0.707	[0.544, 0.814]
PTSD	3.807	0.749	2.404	0.855	−1.403	–	–	−5.975	<0.001	0.871	[−0.871,-0.871]

NB: Δ = T2 − T1. Negative Δ indicates symptom reduction (Anxiety/Depression/PTSD); positive Δ indicates increase (Resilience).

The paired sample *t*-test results showed that pre-test anxiety scores (*M* = 3.489, SD = 0.718) were significantly higher than post-test anxiety scores (*M* = 2.648, SD = 0.736). This indicates that school-based regular aerobic exercise intervention has a significant impact on reducing anxiety symptoms among college students (*t* = −6.352, *p* < 0.001, Cohen’s *d* = −0.926, 95% CI = [−1.266, −0.580]). The effect size far exceeds the threshold of 0.8, indicating a very large effect of the intervention on improving anxiety symptoms.

Pre-test depression scores (*M* = 3.514, SD = 0.647) were significantly higher than post-test depression scores (*M* = 3.087, SD = 0.383). This suggests that school-based regular aerobic exercise intervention significantly impacts depression symptoms in college students (*t* = −3.963, *p* < 0.001, Cohen’s *d* = −0.578, 95% CI = [−0.885, −0.266]). The effect size falls within the medium range, suggesting a notable improvement effect on depression symptoms.

Wilcoxon signed-rank test results indicated that pre-test psychological resilience scores (*M* = 2.843, SD = 0.691) were significantly lower than post-test psychological resilience scores (*M* = 3.585, SD = 0.534). This indicates that school-based regular aerobic exercise intervention has a significant effect on enhancing psychological resilience among college students (*Z* = 4.842, *p* < 0.001, *r* = 0.707, 95% CI = [0.544, 0.814]). Pre-test PTSD scores (*M* = 3.807, SD = 0.749) were significantly higher than post-test scores (*M* = 2.404, SD = 0.855), indicating that school-based regular aerobic exercise intervention can effectively improve PTSD symptoms (*Z* = −5.975, *p* < 0.001, *r* = 0.871, 95% CI = [−0.871,–0.871]).

### 3.5 Clinical significance analysis

To complement the statistical significance findings, we examined the clinical meaningfulness of observed changes by analyzing categorical shifts in symptom severity and the proportion of participants crossing established clinical thresholds.

#### 3.5.1 PTSD clinical recovery

Using the established clinical cutoff score of 53 on the PTSD Checklist for DSM-5 (PCL-5), which indicates probable PTSD diagnosis, we examined the proportion of participants who transitioned from above to below this threshold following the intervention. All 47 participants met the inclusion criterion of PCL-5 ≥ 53 at baseline, confirming probable PTSD. Following the 21-day exercise intervention, 27 participants (57.45%) scored below the clinical cutoff, indicating clinically significant improvement and potential diagnostic remission. The remaining 20 participants (42.55%) continued to score above the cutoff, though all showed some degree of symptom reduction. Table 5 presents the distribution of participants relative to the clinical cutoff at both time points.

**Table 5:** Ptsd clinical cutoff analysis (*N* = 47).

PCL-5 status	Pre-test [*n* (%)]	Post-test [*n* (%)]
Above cutoff (≥53): Probable PTSD	47(100%)	20 (42.55%)
Below cutoff (<53): Non-clinical	0(0%)	27 (57.45%)

Additionally, we calculated the Reliable Change Index (RCI) to determine whether individual changes exceeded measurement error (55). Using the standard error of measurement derived from the scale’s reliability (Cronbach’s *α* = 0.939 at pre-test), 41 participants (87.23%) demonstrated reliable improvement (RCI > 1.96), indicating that their symptom reduction was unlikely due to measurement error alone. Six participants (12.77%) showed improvement that did not exceed the threshold for reliable change, and no participant showed reliable deterioration.

#### 3.5.2 Anxiety severity category shifts

Based on the severity classification criteria for the Self-Rating Anxiety Scale, participants were categorized into four groups: no anxiety (<0.60), mild to moderate anxiety (0.60–0.72), moderate to severe anxiety (0.72–0.84), and severe anxiety (>0.84). Table 6 presents the cross-tabulation of anxiety severity categories from pre-test to post-test.

**Table 6:** Anxiety severity category changes from pre-test to post-test.

Pre-test category	Post-test Category				Total
	No anxiety	Mild–moderate	Moderate–severe	Severe	
No anxiety	5	1	0	0	6 (12.77%)
Mild–moderate	15	6	1	1	23 (48.94%)
Moderate–severe	7	2	0	0	9 (19.15%)
Severe	3	3	3	0	9 (19.15%)
Total post-test	30 (63.83%)	12 (25.53%)	4 (8.51%)	1 (2.13%)	47(100%)

At baseline, 41 participants (87.23%) met criteria for clinical anxiety (severity index ≥ 0.60), including 23 (48.94%) in the mild–moderate range, 9 (19.15%) in the moderate–severe range, and 9 (19.15%) in the severe range. Following the intervention, 17 participants (36.17%) remained in clinical anxiety categories, representing a substantial reduction in clinical cases.

Examination of category transitions revealed that 36 participants (76.60%) improved by at least one severity category, 8 (17.02%) remained in the same category, and 3 participants (6.38%) moved to a more severe category. Among those with clinical anxiety at baseline, 25 of 41 (60.98%) transitioned to the non-clinical range (no anxiety) following the intervention.

#### 3.5.3 Depression severity category shifts

Using the same severity classification approach for the Self-Rating anxiety Scale, participants were categorized based on their severity index scores. Table 7 presents the depression severity category transitions.

**Table 7:** Depression severity category shifts.

Pre-test category	Post-test category				Total
	No depression	Mild–moderate	Moderate–severe	Severe	
No depression	3	0	0	0	3 (6.38%)
Mild–moderate	6	17	1	1	25 (53.19%)
Moderate–severe	3	9	0	0	12 (25.53%)
Severe	2	5	3	0	7 (14.89%)
Total Post-test	14 (29.79%)	31 (65.96%)	1 (2.13%)	1 (2.13%)	47 (100%)

At baseline, 44 participants (93.62%) met criteria for clinical depression (severity index ≥ 0.60), including 25 (53.19%) in the mild–moderate range, 12 (25.53%) in the moderate–severe range, and 7 (14.89%) in the severe range. Following the intervention, 33 participants (70.21%) remained in clinical depression categories, representing a notable reduction in clinical cases.

Examination of category transitions revealed that 25 participants (53.19%) improved by at least one severity category, 20 (42.55%) remained in the same category, and only 2 participants (4.26%) moved to a more severe category. Among those with clinical depression at baseline, 11 of 44 (25.00%) transitioned to the non-clinical range (no depression) following the intervention. Notably, all 19 participants (100%) who were in the moderate–severe or severe range at baseline improved to less severe categories, with none remaining in these highest severity categories post-intervention.

The clinical indicators are shown in Table 8.

**Table 8:** Summary of clinical significance indicators.

Indicator	PTSD	Anxiety	Depression
Pre-test clinical cases	47(100%)	41(87.23%)	44(93.62%)
Post-test clinical cases	20(42.55%)	17(36.17%)	33(70.21%)
Remission rate	27(57.45%)	25(60.98%)	11(25.00%)
Category improvement	–	36(76.60%)	25(53.19%)
No change in category	–	8(17.02%)	20(42.55%)
Category worsening	–	3(6.38%)	2(4.26%)
Reliable change (RCI > 1.96)	41(87.23%)	–	–

NB: Remission rate calculated as proportion of baseline clinical cases who transitioned to non-clinical status: PTSD (27/47), Anxiety (25/41), Depression (11/44).

### 3.6 Exploratory change-score associations

#### 3.6.1 Preliminary analyses

Before conducting the formal mediation analysis, we calculated change scores for all study variables, as shown in Table 9. Positive values of *Δ*Resilience indicate increased resilience, whereas negative values of ΔAnxiety, ΔDepression, and ΔPTSD indicate decreased symptoms. This suggests that 21 days of regular exercise can effectively improve the mental health of college students with symptoms of PTSD.

**Table 9:** Scores for changes in study variables.

Variables	Mean Change (Δ = T2-T1)	SD	Min	Max
Δ Psychological resilience	0.742	0.846	−0.44	2.72
Δ Anxiety	−0.842	0.908	−2.00	3.10
Δ Depression	−0.427	0.738	−2.30	1.60
Δ PTSD	−1.403	0.311	−2.00	−0.85

#### 3.6.2 Exploratory change-score analysis

Given the single-group pre-post design, we conducted exploratory regression analyses to examine whether individual differences in resilience change (*Δ* resilience) were associated with individual differences in symptom changes (Δ anxiety, Δ depression, and *Δ*PTSD), as shown in Table 10. These analyses explore potential mechanisms but cannot establish causality due to the absence of a control group and the correlational nature of change-score associations.

**Table 10:** Change-score regression results for exploratory analysis.

Outcomes (Δ = post − pre)	Predictors	B	β	SE	t	p	95%CI
Δ Anxiety	Δ resilience	−0.36	−0.335	0.151	−2.385	0.021	[−0.056,-0.663]
Δ Depression		−0.202	−0.231	0.127	−1.592	0.118	[−0.456,0.053]
Δ PTSD		−0.121	−0.329	0.052	−2.336	0.024	[−0.225,-0.017]

NB: Δ represents change scores calculated as post-test minus pre-test values. Negative B coefficients indicate that greater increases in resilience are associated with greater decreases in symptoms.

Anxiety. The change-score regression revealed that Δ resilience significantly predicted Δ anxiety (*β* = −0.335, *p* = 0.021). Participants who showed greater increases in psychological resilience tended to show greater reductions in anxiety symptoms. The model explained approximately 11.2% of the variance in anxiety change (*R*^2^ = 0.112).

Depression. Δ resilience did not significantly predict Δ depression (*β* = −0.231, *p* = 0.118), although the direction of the association was consistent with expectations. This suggests that individual differences in resilience change were not reliably associated with individual differences in depression change in this sample.

PTSD. Δ resilience significantly predicted ΔPTSD (*β* = −0.329, *p* = 0.024). Participants demonstrating greater improvements in psychological resilience showed correspondingly greater reductions in PTSD symptoms. The model explained approximately 10.8% of the variance in PTSD change (*R*^2^ = 0.108).

### 3.7 Cautions for exploratory findings

These exploratory analyses suggest that changes in psychological resilience may be associated with changes in anxiety and PTSD symptoms following the exercise intervention. Specifically, participants who experienced greater enhancement in resilience also tended to experience greater symptom relief.

First, due to the single-group design, we cannot determine whether the intervention caused the observed changes or whether these changes would have occurred naturally over time. Second, the change-score correlations do not establish temporal precedence—we cannot determine whether resilience changes preceded symptom changes or vice versa. Third, the modest effect sizes (*R*^2^ ranging from 0.05 to 0.11) indicate that resilience changes account for only a small proportion of the variance in symptom changes, suggesting that other unmeasured factors likely play important roles.

These findings should therefore be considered hypothesis-generating rather than confirmatory and warrant replication in randomized controlled trials with appropriate temporal measurement of mediators and outcomes.

## 4 Discussion

This study used a single-group pre–post design without randomization or a control condition; therefore, the observed symptom changes cannot be attributed causally to the exercise check-in program. Alternative explanations include natural recovery over time, regression to the mean (given elevated baseline screening scores), repeated-measurement effects, seasonal or academic-calendar influences, and other concurrent supports or lifestyle changes during the 21-day period. Accordingly, the findings should be interpreted as preliminary and hypothesis-generating evidence of within-participant change associated with program participation rather than evidence of efficacy.

Within these constraints, participants who completed both assessments showed pre–post reductions in PTSD symptoms and comorbid anxiety and depression, along with increased resilience. These patterns are consistent with prior work linking physical activity to mental health outcomes, and they support the feasibility of testing brief, scalable exercise-based programs for students with elevated PTSD symptoms in more rigorous designs.

### 4.1 Single group pre-and post-test analysis

The results of the single group pre-and post-test analysis indicate that regular aerobic exercise significantly promotes the psychological health of participants. Engaging in school-based regular aerobic exercise intervention provides college students an immediate sense of control, with physiological responses (like adjusted breathing and increased heart rate) and pleasurable experiences serving as positive feedback (56). This accumulation of positive experiences continually strengthens the individual’s motivation to exercise and adherence to the behavior. Consequently, it effectively breaks the passive and helpless feelings that trauma survivors may develop in daily life, helping them reconstruct a positive self-schema through specific exercise practices.

Regular exercise tasks require students to complete activities at fixed times or in specific contexts, thus providing a structured coherence to their lives (57). For college students experiencing psychological distress, having a structured time and behavior pattern significantly reduces the intrusion of random stressors and avoids the psychological emptiness that may arise from a lack of goals and planning. More critically, this rhythmic arrangement enables students to gradually experience the transition from chaos to order, enhancing their sense of stability and security. Security buffers subjective feelings of external threat and diminishes excessive vigilance toward the environment (58).

It is noteworthy that regular aerobic exercise also serves as a significant opportunity for emotional transformation. After completing exercise, individuals typically experience feelings of joy, relaxation, and even a slight sense of accomplishment, introducing new variables into previously rigid negative emotional patterns (59). Individuals under constant stress often tend to interpret external events negatively, leading to cognitive rigidity (60). Exercise provides a means to interrupt this pattern, allowing them to gain psychological relief from brief emotional peaks (61). As these positive experiences accumulate, individuals’ cognitive flexibility gradually increases, manifested through more open thinking, adaptive self-talk, and positive meaning construction.

### 4.2 Clinical meaningfulness of observed changes

The present study demonstrated not only statistically significant improvements across all three primary outcome measures, but also clinically meaningful changes that underscore the therapeutic value of the online group intervention for earthquake-affected adolescents.

Regarding PTSD symptomatology, the intervention produced substantial clinical benefits. At baseline, all 47 participants met the clinical threshold for probable PTSD, reflecting the severe psychological impact of earthquake trauma on this population. Following the intervention, 27 participants (57.45%) transitioned below the clinical cutoff, indicating diagnostic-level remission. Furthermore, reliable change index analysis revealed that 41 participants (87.23%) exceeded the threshold for reliable improvement (RCI > 1.96), demonstrating that the observed reductions in PTSD symptoms were not attributable to measurement error. The mean reduction of 18.36 points on the PCL-5 substantially exceeded the established minimal clinically important difference of 10–20 points, suggesting that participants experienced perceptible and meaningful relief from intrusive memories, avoidance behaviors, negative cognitions, and hyperarousal symptoms.

For anxiety symptoms, the clinical impact was equally pronounced. Among the 41 participants who met criteria for clinical anxiety at baseline, 25 (60.98%) achieved remission to non-clinical status following the intervention. Category-level analysis further demonstrated that 36 participants (76.60%) improved by at least one severity category, with only 3 participants (6.38%) showing category-level worsening. Notably, of the 14 participants classified in the moderate–severe or severe anxiety ranges at baseline, 13 (92.86%) improved to less severe categories, with the number of severe cases decreasing from 6 to zero. These patterns indicate that the intervention was particularly effective for those with more pronounced anxiety presentations.

Depression outcomes similarly reflected meaningful clinical improvement, though the pattern differed somewhat from the other measures. Of the 44 participants meeting clinical criteria for depression at baseline, 11 (25.00%) transitioned to the non-clinical range. While this remission rate was lower than those observed for PTSD and anxiety, category-level analysis revealed that 25 participants (53.19%) improved by at least one severity category, with only 2 (4.26%) showing worsening. Particularly noteworthy was the complete resolution of severe depression cases: all 19 participants who were in the moderate–severe or severe depression categories at baseline improved to less severe categories, with no participants remaining at these highest severity levels post-intervention.

Collectively, these findings indicate that the online group intervention produced clinically meaningful benefits across the spectrum of trauma-related psychopathology. The convergence of multiple indicators—including remission rates, reliable change indices, and severity category transitions—provides robust evidence that the observed statistical effects translated into tangible improvements in participants’ psychological functioning. The particularly strong effects observed for PTSD symptoms align with the trauma-focused nature of the intervention, while the substantial improvements in anxiety and depression suggest beneficial effects on the broader constellation of post-disaster psychological distress.

### 4.3 Exploratory analysis of resilience as a potential mechanism

The exploratory change-score analyses provide preliminary evidence suggesting that improvements in psychological resilience may be associated with reductions in anxiety and PTSD symptoms following the exercise intervention. Specifically, participants who showed greater increases in resilience also tended to show greater decreases in anxiety (*β* = −0.335, *p* = 0.021) and PTSD symptoms (*β* = −0.329, *p* = 0.024). These associations, while modest in magnitude, are consistent with theoretical frameworks positing resilience as a dynamic psychological resource that influences trauma recovery (62).

However, it is crucial to interpret these findings within the constraints of our study design. The single-group pre-post design precludes causal inference, as we cannot rule out alternative explanations for the observed associations. The correlational nature of change-score analyses means that we cannot determine temporal precedence—whether resilience improvements preceded symptom relief, occurred simultaneously, or followed symptom changes. Additionally, third variables not measured in this study may account for the observed associations between resilience and symptom changes.

The non-significant association between resilience change and depression change (*β* = −0.231, *p* = 0.118) warrants consideration. While the direction of the effect was consistent with hypotheses, the lack of statistical significance may reflect insufficient statistical power given our sample size, different mechanisms underlying depression versus anxiety and PTSD, or the possibility that resilience operates differently on depression within the context of exercise interventions. Future studies with larger samples and more frequent measurement occasions could help clarify these relationships.

Despite these limitations, the findings provide a foundation for hypothesis development regarding the mechanisms through which exercise interventions may affect psychological outcomes. The consistent pattern of associations between resilience enhancement and symptom reduction across anxiety and PTSD outcomes suggests that resilience-building components may be valuable targets for optimization in future intervention designs. These findings align with conservation of resources theory (Alvaro et al., 2010), which suggests that resource acquisition through exercise may buffer against the resource depletion associated with trauma exposure.

### 4.4 Practical implications

These findings carry several implications for practice. First, schools should consider integrating brief, structured exercise opportunities into daily routines as a component of mental health promotion. The online delivery format used in this study demonstrates feasibility for implementation without requiring specialized facilities or extensive personnel training. Schools might incorporate guided exercise sessions into morning routines, physical education classes, or after-school programming, with particular attention to students showing elevated depressive symptoms.

Second, the accessibility of the exercise modalities studied—jump rope, running, and Tai Chi—suggests potential for broad implementation. These activities require minimal equipment, can be performed in limited spaces, and are adaptable to varying fitness levels. The flexibility to choose and switch between modalities enhanced acceptability in our sample and may be an important design feature for real-world programs. Mandating a single exercise type may be less effective than offering structured choice within defined parameters.

Third, the online platform approach offers scalability advantages, particularly for reaching adolescents in under-resourced areas or those who face barriers to in-person programming. However, our attrition rate indicates that online delivery alone is insufficient; the combination of digital content with human support (facilitator check-ins, personalized feedback) was likely important for maintaining engagement. Future implementations should preserve this blended approach rather than relying solely on automated content delivery.

Fourth, the 21-day duration, while sufficient to produce measurable effects, is brief relative to the chronic nature of adolescent depression. Programs should plan for maintenance and follow-up support to sustain initial gains. This might include transitioning participants to ongoing exercise opportunities within schools or communities, periodic booster sessions, or peer support networks that encourage continued physical activity.

Finally, exercise interventions should be positioned as complementary to, rather than substitutes for, established mental health treatments. Adolescents with moderate-to-severe depression or active suicidal ideation require professional clinical care; exercise may serve as an adjunct that enhances treatment response or as a preventive strategy for those with subclinical symptoms.

### 4.5 Safety considerations and exercise tolerance

The withdrawal of 15 participants due to adverse physical symptoms represents a significant concern that warrants careful examination. When considered alongside the finding that non-completers exhibited significantly higher baseline PTSD, anxiety, and depression scores, a troubling pattern emerges: the intervention may be least tolerable for those who are most symptomatic and potentially most in need of treatment.

Several mechanisms may explain the elevated vulnerability of more symptomatic participants to exercise-induced adverse effects. First, individuals with elevated PTSD and anxiety often exhibit chronic hyperarousal, including elevated resting heart rate, heightened sympathetic nervous system activation, and dysregulated stress response systems. The additional physiological demands of exercise may have compounded this existing arousal, potentially triggering symptoms such as dizziness, lightheadedness, or panic-like sensations. Second, trauma-affected individuals frequently demonstrate altered interoceptive processing, with some experiencing hypervigilance to bodily sensations that may amplify the perceived intensity of normal exercise-related experiences (e.g., increased heart rate, sweating, breathlessness) and trigger trauma-associated fear responses. Third, elevated depression is associated with physical deconditioning, fatigue, and reduced exercise tolerance, potentially rendering standard exercise intensities more challenging for these individuals. Fourth, lower psychological resilience may reduce capacity to persist through physical discomfort that more resilient individuals might tolerate.

The concentration of symptom-related dropouts in the first 2 weeks suggests that the initial adaptation period poses the greatest risk. This finding has direct implications for protocol design: more gradual introduction of exercise, with extended low-intensity acclimation periods, may be essential for more symptomatic participants.

These findings do not necessarily contraindicate exercise-based interventions for trauma-affected populations. Indeed, substantial evidence supports the benefits of exercise for PTSD and related conditions. However, our results suggest that standard exercise protocols may require significant modification for individuals with more severe symptomatology. Recommendations for future implementation include: Comprehensive pre-intervention assessment of current fitness levels, exercise history, physical health conditions, and symptom severity to identify individuals at elevated risk for adverse responses; Development of differentiated exercise prescriptions based on baseline symptom severity and fitness levels, with lower-intensity starting points for more affected individuals; Longer initial phases with very gradual intensity progression, particularly for participants with elevated symptoms or limited exercise experience.

### 4.6 Research limitations and future directions

Several directions for future research emerge from this study’s findings and limitations. Most urgently, randomized controlled trials with longer follow-up periods are needed to establish whether observed effects persist beyond the immediate post-intervention period and to identify optimal intervention duration for sustained benefit. Our 21-day protocol produced meaningful short-term effects, but the durability of these effects remains unknown.

Future studies should incorporate objective measures of both exercise behavior (e.g., accelerometry) and biological outcomes (e.g., inflammatory markers, cortisol, neuroimaging) to reduce reliance on self-report and illuminate mechanisms of action. Such measures would address the methodological limitations of the present study and provide stronger evidence for causal claims.

Research should examine potential moderators of intervention response. Our sample was insufficiently powered to detect differential effects by sex, baseline symptom severity, exercise history, or exercise modality preference. Identifying which adolescents benefit most—and which may require modified or alternative approaches—would enable more efficient resource allocation.

Implementation research examining real-world adoption, adaptation, and sustainment of exercise-based mental health programming in schools and communities would bridge the gap between controlled research and practical impact. Identifying barriers and facilitators to implementation, cost-effectiveness relative to other mental health interventions, and strategies for maintaining program quality at scale represent important next steps toward translating this evidence into public health benefit.

This study primarily recruited participants from the same university, resulting in a concentration of cultural backgrounds, socioeconomic levels, and campus environments; thus, the generalizability and cross-cultural applicability of the findings still require validation. This bias limits the external validity of the results, which might overestimate the true group effects. Future research should strive for diversity in samples and breakthroughs in cross-cultural effects. Expanding the intervention to adolescents in middle school, workplace populations, or even veterans can allow for comparisons of exercise effects in different developmental tasks and social pressure contexts.

Attrition and selection bias. A substantial proportion of participants did not complete the intervention and post-test assessments. This level of attrition raises concerns about selection bias and limits generalizability. Participants who completed the 21-day check-in may have differed systematically from those who discontinued (e.g., greater exercise tolerance, higher motivation, fewer time constraints, or different baseline symptom severity). Consequently, the magnitude of within-person symptom change observed among completers may not represent the change that would occur in the broader screened population. Future studies should implement strategies to reduce attrition (e.g., graded intensity options, adverse-event monitoring and management, flexible scheduling, and enhanced follow-up procedures) and should examine baseline differences between completers and non-completers, as well as apply analytic approaches that better accommodate missing data (e.g., mixed-effects models and/or multiple imputation), to strengthen the robustness of conclusions.

Importantly, the present findings should be interpreted in light of the single-group pre-post design. Symptom reductions and resilience gains may reflect multiple alternative explanations, including regression to the mean, spontaneous symptom fluctuation and natural recovery over time, repeated-measurement effects, seasonal or contextual changes during the study period, or concurrent informal support. Therefore, the results provide preliminary evidence of feasibility and within-person change, but they do not demonstrate causal effectiveness. Future studies should employ randomized controlled designs (e.g., waitlist or active control conditions), objective activity monitoring (e.g., wearables), and longer-term follow-up to test durability and to strengthen causal inference regarding mechanisms.

The substantial attrition rate and the systematic differences between completers and non-completers represent significant threats to internal and external validity. Of the 83 participants with complete baseline data, 36 (43.37%) did not complete the intervention. Independent samples *t*-tests revealed that non-completers exhibited significantly higher baseline levels of PTSD, anxiety, and depression, as well as significantly lower psychological resilience, compared to completers. This pattern of differential attrition indicates that the intervention may have been less acceptable, tolerable, or accessible to those with more severe psychopathology and fewer psychological resources—precisely the individuals who may be in greatest need of intervention. The implications of this attrition pattern are substantial. The final analytic sample likely represents a more resilient, less symptomatic subgroup of the initially enrolled population, potentially inflating effect size estimates and remission rates. The true efficacy of the intervention for adolescents with more severe trauma-related symptoms remains uncertain. Clinicians and researchers considering implementation of similar interventions should be aware that individuals presenting with higher symptom severity and lower resilience may require additional support, modified protocols, or alternative treatment approaches to enhance retention and benefit from exercise-based interventions.

Beyond the measured baseline differences, self-selection processes may have introduced additional unmeasured confounds. Participants who volunteered for an exercise-based intervention and persisted through completion may have possessed more positive attitudes toward physical activity, greater self-efficacy for behavior change, stronger family support, or other characteristics associated with favorable treatment response. These unmeasured factors may further limit generalizability to the broader population of earthquake-affected adolescents.

Due to the single-group pre-post design, we employed change-score regression analyses. While this approach is transparent and appropriate for our design, it cannot establish that resilience changes causally mediate intervention effects on symptoms. The change-score correlations observed may reflect shared method variance, common causes affecting both resilience and symptom changes, or reverse causation (symptom improvement leading to resilience enhancement rather than vice versa). Future research employing randomized controlled trials with multiple measurement waves would enable more rigorous mediation analysis using appropriate statistical techniques such as latent change score models or multilevel structural equation modeling, which can better account for measurement error and establish temporal precedence of mediators relative to outcomes.

## 5 Conclusion

This study verified the intervention potential of regular aerobic exercise on college students’ post-traumatic stress disorder and its accompanying symptoms in a real campus environment. The results indicate that exercise can significantly alleviate negative emotions such as anxiety and depression while promoting the enhancement of psychological resilience, thereby providing internal psychological resource support for trauma recovery. Model analysis further reveals that psychological resilience plays a partial mediating role in the relationship between exercise and symptom relief, indicating that the intervention effects stem not only from physiological activation but also from the reconstruction and reinforcement of internal adaptability. This finding provides new theoretical evidence for the integration of exercise psychology and trauma intervention. The significant implications of this research lie in providing a low-cost, easily disseminated, and low-stigma non-pharmacological intervention program for campus mental health service systems. By encouraging students to incorporate exercise into their daily lives, the prevalence and accessibility of mental health services can be effectively enhanced, achieving both preventive and intervention goals. However, the study also has limitations in design and sampling, highlighting the need for more rigorous experimental designs, broader sample sources, and more comprehensive assessment tools in future research. Overall, this research enriches the theoretical framework regarding the relationship between exercise and mental health, while providing reliable evidence for universities to formulate mental health promotion policies. Future efforts to integrate longitudinal follow-up, multidisciplinary measurement, and cross-cultural comparisons will enhance understanding of the mechanisms involved and promote regular aerobic exercise as an essential component of campus mental health service systems.

## Data Availability

The raw data supporting the conclusions of this article will be made available by the authors, without undue reservation.
